# Factors predicting one-year post-surgical mortality amongst older Asian patients undergoing moderate to major non-cardiac surgery – a retrospective cohort study

**DOI:** 10.1186/s12893-019-0654-x

**Published:** 2020-01-13

**Authors:** Lydia Q. Liew, Wei Wei Teo, Edwin Seet, Lyn Li Lean, Ambika Paramasivan, Joanna Tan, Irene Lim, Jiexun Wang, Lian Kah Ti

**Affiliations:** 1 Department of Anaesthesia, National University Health System, 5 Lower Kent Ridge Road, Singapore, 119074 Singapore; 2 Department of Anaesthesia, Khoo Teck Puat Hospital, 90 Yishun Central, Singapore, 768828 Singapore

**Keywords:** Mortality, Post-surgery, Asian, Anaemia, BMI

## Abstract

**Background:**

While short-term perioperative outcomes have been well studied in Western surgical populations, the aim of this study is to look at the one-year perioperative mortality and its associated factors in an Asian surgical population after non-cardiac surgery.

**Methods:**

A retrospective cohort study of 2163 patients aged above 45 undergoing non-cardiac surgery in a university-affiliated tertiary hospital from January to July 2015 was performed. Relevant demographic, clinical and surgical data were analysed to elicit their relationship to mortality at one year after surgery. A univariate analysis was first performed to identify significant variables with *p*-values ≤ 0.2, which were then analysed using Firth multiple logistic regression to calculate the adjusted odds ratio.

**Results:**

The one-year mortality in our surgical population was 5.9%. The significant factors that increased one-year mortality include smoking (adjusted OR 2.17 (1.02–4.45), *p* = 0.044), anaemia (adjusted OR 1.32 (1.16–1.47), *p* < 0.001, for every 1 g/dL drop in haemoglobin level), lower BMI (adjusted OR 0.93 (0.87–0.98), *p* = 0.005, for every 1 point increase in BMI), Malay and Indian ethnicity (adjusted OR 2.68 (1.53–4.65), *p* = 0.001), peripheral vascular disease (adjusted OR 4.21 (1.62–10.38), *p* = 0.004), advanced age (adjusted OR 1.04 (1.01–1.06), p = 0.004, for every one year increase in age), emergency surgery (adjusted OR 2.26 (1.29–3.15), p = 0.005) and malignancy (adjusted OR 3.20 (1.85–5.52), p < 0.001).

**Conclusions:**

Our study shows that modifiable risk factors such as malnutrition, anaemia and smoking which affect short term mortality extend beyond the immediate perioperative period into longer term outcomes. Identification and optimization of this subset of patients are therefore vital. Further similar large studies should be done to develop a risk scoring system for post-operative long-term outcomes. This would aid clinicians in risk stratification, counselling and surgical planning, which will help in patients’ decision making and care planning.

## Background

More than 200 million adults undergo non-cardiac surgeries every year [1]. With recent advancements in medicine, older patients with potentially higher burdens of heart disease and other comorbidities are surviving longer and go on to develop conditions that require surgical intervention. The shift in practice to offer surgical intervention for increasingly complex and older patients may potentially increase perioperative mortality rates in spite of advances in surgical methods.

There are many studies that look into 30-day perioperative outcomes including all-cause mortality, but while 30-day perioperative mortality is undeniably an important marker for immediate surgical complications and may guide postoperative resource allocation, longer term survival may ultimately be more meaningful to patients and may have more relevance when quoting risks to patients and their family members, allowing patients to make more informed decisions regarding their care and treatment.

One-year perioperative mortality has been reported to be up to 6–8% in non-cardiac surgery and [1, 2] preoperative chronic heart and lung disease have predictably been shown to increase one-year perioperative mortality in patients undergoing cardiac surgery [3]. However, there is a paucity of studies investigating factors associated with the longer-term mortality among non-cardiac surgical patients. Furthermore, most large studies have been done on Western populations, limiting applicability to Asian populations like ours which have different genetic characteristics, possibly resulting in differing risk factors and mortality rates [4].

Hence, the aim of this study is to look at factors associated with increased one-year mortality after non-cardiac surgery enabling us to identify high risk groups of patients, which would allow effective patient selection, risk stratification, appropriate patient counselling, surgical planning and preoperative optimization.

## Methods

Following ethics committee approval from the Institutional Review Board (National Healthcare Group Domain Specific Review Board reference: 2016/01273), a retrospective cohort study of 2300 patients aged 45 years old and above undergoing non-cardiac surgery from January 2015 to July 2015 was performed in a university-affiliated tertiary hospital.

Patients undergoing surgery were identified through operating theatre audit records which capture > 90% of patients undergoing any form of surgery. Members of the research team used International Classification of Diseases coding and manually reviewed each patient’s electronic medical records to obtain relevant information pertaining to their baseline characteristics, co-morbidities and nature of surgery.

The inclusion criteria included patients 45 years old and above undergoing intermediate or high-risk non-cardiac surgery, defined as surgery requiring at least 23 h stay in hospital. Patients undergoing multiple surgeries had only the index surgery considered, with their subsequent surgeries excluded from the analysis, resulting in a total of 2163 patient encounters analysed.

Relevant demographic, clinical and surgical data were analysed to elicit their relationship to mortality at one year after surgery. The presence or absence of patient comorbidities were considered positive if they were known to be present at the time of surgery as indicated by anaesthetic records. These records are routinely obtained by direct patient questioning as well as by searching electronic hospital records. Laboratory investigations were analysed if they were performed within six months prior to surgery with no change in patient’s medical status in between. If there were more than one of the same investigation performed in the same patient, the one done closest to the time of surgery was used for analysis. All perioperative factors studied are shown in Appendix Table 4. Death was determined using electronic medical records that is linked to our national health registry. This registry includes information from all primary healthcare services and hospitals in Singapore. If the patient has deceased, this would be indicated on the record, including the date of death. Proof that a patient was alive was confirmed by searching for evidence of any subsequent healthcare visits, prescriptions, laboratory and radiological investigations within the time period.

All statistical analyses were done using IBM SPSS version 25.0 (Armonk, NY, USA) and R version 3.4.4. This manuscript adheres to the applicable STROBE guidelines.

To find significant perioperative variables to account for mortality, a univariate analysis was first performed. Categorical data were analysed using the chi-squared test and continuous data were analysed using the 2-sample t test. Significant variables were identified as those having a *p*-value of < 0.2, which were then analysed using Firth multivariate logistic regression to calculate the adjusted odds ratio. These variables included age, ethnicity, ASA, gender, BMI, emergency surgery, John Hopkin’s classification, type of anaesthesia, duration of surgery, haemoglobin level, glomerular filtration rate, urea level, and presence of the following – congestive cardiac failure, ischaemic heart disease, valvular heart disease, peripheral vascular disease, other cardiovascular disease, smoking, respiratory disease, previous stroke or transient ischaemic attack, endocrine disease, liver disease, haematological or coagulation disorders, malignancy. Continuous variables such as BMI and haemoglobin level were also analysed categorically, based on the World Health Organisation’s severity classification. Significant factors were identified as those having a *p*-value of < 0.05.

## Results

Patient demographics and surgical characteristics are shown in Tables 1 and 2 respectively. A total of 2163 patients were studied. Patients with missing data were excluded in the multivariate analysis, resulting in the analysis being performed on 2138 patients. The population consisted of 53.4% male. 70.5% of patients were of Chinese ethnicity, 19% were Malays, 8.2% were Indians, which closely reflects the ethnic distribution in Singapore [5]. Majority of the population were ASA 2 (47.4%) and 3 (44.3%), and 28.3% underwent emergency surgery. There was a wide range of surgeries across different disciplines and most (> 80%) were done under general anaesthesia.

**Table 1:**
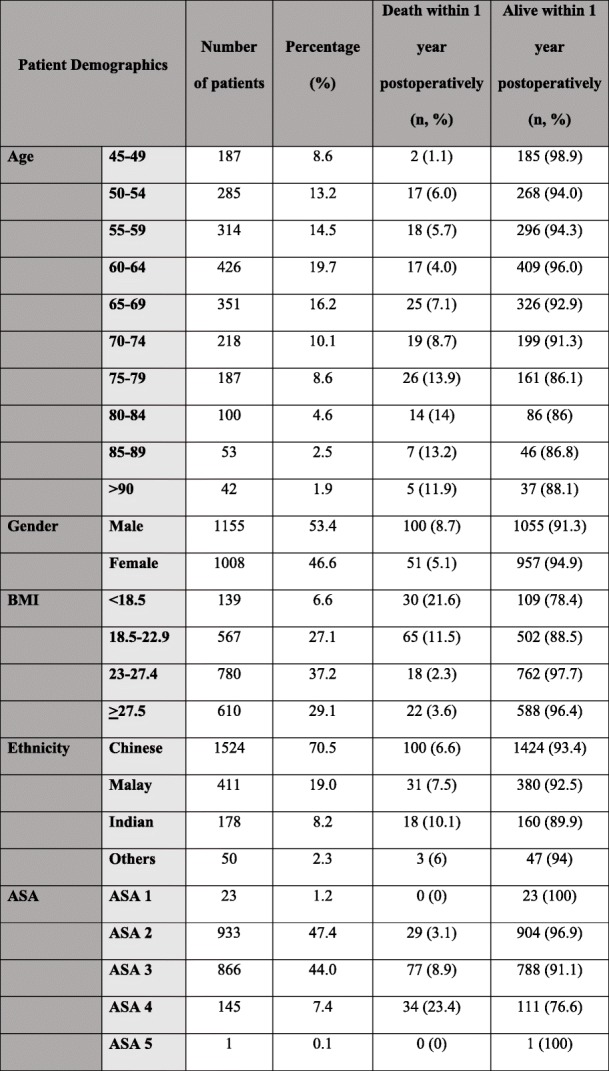
Table showing demographics of patient population studied

**Table 2:**
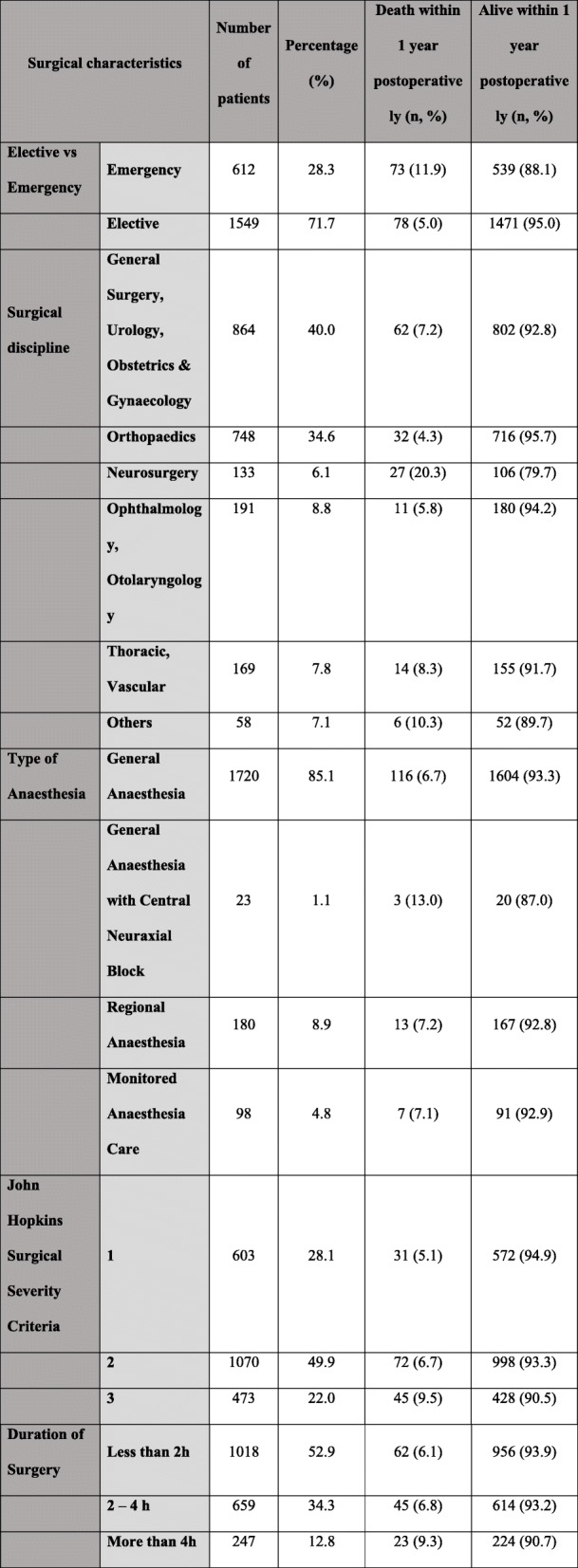
Table showing surgical characteristics of the population studied

The one-year mortality in our surgical population was 5.9% (*n* = 127). All factors studied are shown in Table 4, found in the Appendix. The significant factors that increased one-year mortality are shown in Table 3.

**Table 3:**
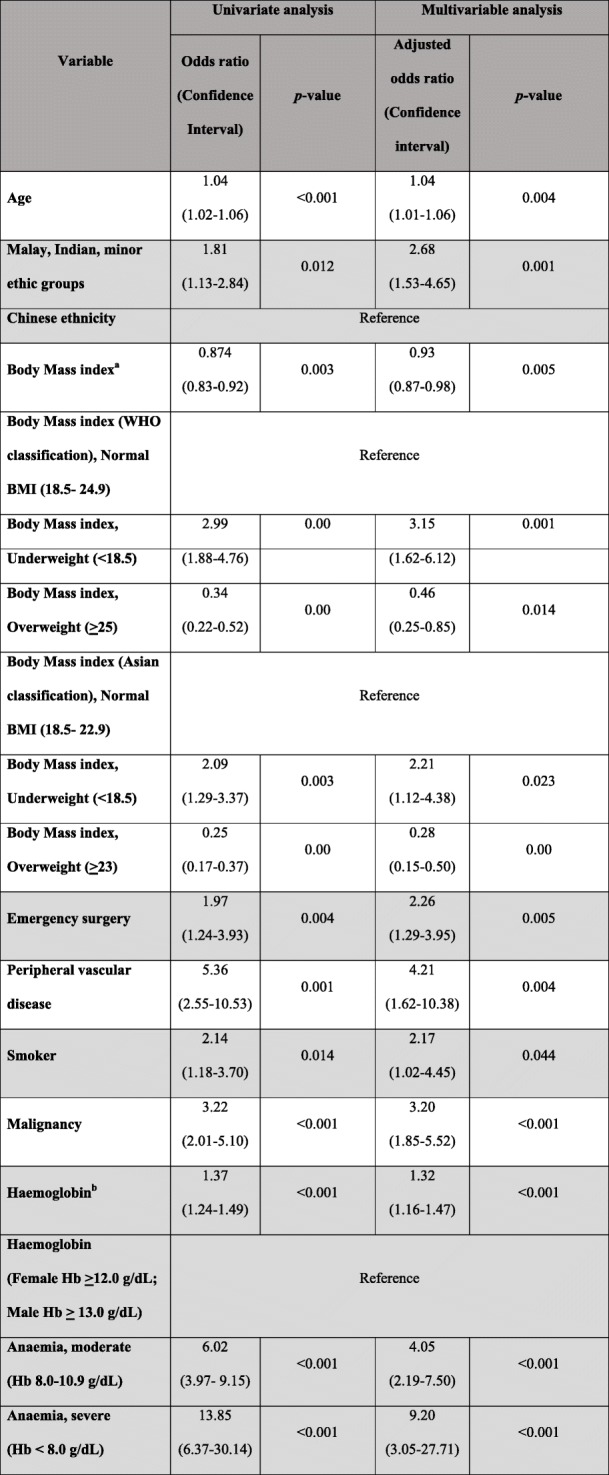
Table showing significant variables associated with 1-year mortality in both univariate and multivariate regression models

^a^For every 1 point increase in BMI

^b^For every 1g/dL drop in haemoglobin

The significant factors that increased one-year mortality include smoking (adjusted OR 2.17 (1.02–4.45), *p* = 0.044), anaemia which was analysed both continually (adjusted OR 1.32 (1.16–1.47), *p* < 0.001, for every 1 g/dL drop in haemoglobin level), as well as categorically (adjusted OR for moderate anaemia 4.05 (1.16–1.47), *p* < 0.001; adjusted OR for severe anaemia 9.20 (3.05–27.71), p < 0.001)), lower BMI which was analysed both continually (adjusted OR 0.93 (0.87–0.98), *p* = 0.005, for every 1 point increase in BMI), as well as categorically (adjusted OR 3.15 (1.62–6.12), *p* = 0.002), Malay and Indian ethnicity (adjusted OR 2.68 (1.53–4.65), *p* = 0.001), peripheral vascular disease (adjusted OR 4.21 (1.62–10.38), *p* = 0.004), advanced age (adjusted OR 1.04 (1.01–1.06), p = 0.004 for every one year increase in age), emergency surgery (adjusted OR 2.26 (1.29–3.15), p = 0.005) and malignancy (adjusted OR 3.20 (1.85–5.52), p < 0.001).

## Discussion

The one-year postoperative mortality in our study population is comparable to the rates published in most international studies [1, 2]. The factors that have been shown to have a significant impact on one-year mortality rates are outlined below.

### Anaemia

The effect of anaemia on 30-day and in-hospital mortality has been extensively studied. A large retrospective study involving 39,309 patients in the United Kingdom found that the prevalence of anaemia was 31.1% in males and 26.5% in females and that patients with preoperative anaemia had a higher in-hospital mortality compared with patients without anaemia [6]. Similar large studies highlighting the detrimental effects of preoperative anaemia on perioperative outcomes have led to the development of guidelines for perioperative management of anaemia [7]. Our study has shown that anaemia, particularly moderate and severe anaemia, not only affects short-term mortality after surgery but extends to one-year mortality as well, further emphasizing the importance of management of anaemia perioperatively especially given its high prevalence and modifiability.

We suggest that a multimodal, multidisciplinary blood management program should be implemented in which all surgical patients should be evaluated as early as possible to optimize patient haemoglobin and iron stores and that elective surgeries should be scheduled in a way that allows for such optimization. Further studies should be done to ascertain whether it is anaemia alone or any resultant transfusion that increases adverse outcomes [8].

Transfusion has its risks including transfusion reactions, infections and metabolic complications. Hence, the decision to transfuse should be considered in a case to case basis taking into the account the patient’s comorbidities, ongoing bleeding and clinical stability. Many guidelines have since been developed recommending transfusion thresholds in different clinical scenarios [7].

### Malignancy

Patients with known malignancies comprise a significant proportion of our study population (16.1%) and were expectedly shown to have an increased one-year mortality rate. This is a reminder of the impact of the systemic impact of progression of cancer and its treatment on frailty, anaemia, immunosuppression and cardiac depression.

There has been increasing interest in the effect on intraoperative anaesthetic technique and drug choices on long-term outcomes in cancer patients due to interactions with the cellular immune system**.** Preclinical and clinical studies suggest that anaesthetics and adjuvants such as opioids and nitrous oxide given in the perioperative period can affect cancer recurrence and survival, perhaps tipping the balance in some instances to determine if cancer progresses or regresses [9] and some retrospective studies have hinted that regional anaesthesia can play a protective role in cancer surgery [10].

We suggest that the benefits and risks of non-cancer surgery in patients with cancer would need to be sufficiently weighed and discussed extensively. The potential impact of anaesthesia modality and drugs used should be considered when offering surgical intervention to such patients. However, we await larger randomized controlled prospective trials examining the impact of these relationships [10].

### Smoking

Our study validates other small studies that have shown smoking to be an independent risk factor that increases both 30-day and one-year postoperative mortality [11, 12]. International protocols recommend that abstinence from smoking for at least four to eight weeks preoperatively is required for maximal benefit [13–15]. A systematic review with 1194 patients concluded that benefit is seen when intensive smoking cessation interventions in the form of individual counselling and nicotine replacement therapy are applied to patients at least four weeks before surgery and that there is a significant reduction of 30-day postoperative complications including wound healing, respiratory, cardiovascular, urological and other complications requiring treatment [15].

Smaller studies have also shown that interventions are effective in changing smoking behaviour and promoting abstinence postoperatively [16, 17]. A Cochrane review concluded that perioperative smoking cessation was most effective using an intensive intervention program which involved counselling for four to eight weeks [14]. This suggests that smoking cessation should ideally be initiated in surgical clinics the moment elective surgery is proposed in order to minimize perioperative adverse outcomes as well as to use preparation for surgery as an opportune moment to institute positive long-term changes in smoking behaviour.

### Body mass index (BMI)

Interestingly, our results show that a higher BMI is protective for one-year mortality in our surgical population. A meta-analysis done among 30,000 gastric cancer patients which showed that although patients with higher BMIs had longer operation times, increased blood loss and more infective complications, higher BMI had no long term impact on post-operative mortality and long term survival [18]. This is consistent with two meta-analyses which looked into survival amongst the critically ill that showed being overweight conferred a survival advantage [19, 20]. It has been postulated that increased adipose tissue is associated with increased inflammatory mediators, such as leptin and interleukin-10, that may attenuate the inflammatory response and thus potentially improve survival [21]. This may explain the observed mortality advantage among the overweight surgical population. Another study done on 26,908 Mayo Clinic colorectal cancer patients demonstrated a significant disadvantage in overall survival amongst underweight patients as compared to both the normal and overweight patient groups [22]. We believe that it is underweight patients at risk of malnutrition who have a significant survival disadvantage rather than a high BMI actually being protective. This is likely due to nutritional deficiencies causing impairment of immune function, wound healing and attenuation of the body’s metabolic response to stress [23]. Nutritional screening protocols are vital to preoperative evaluation and optimization prior to proposed surgery.

### Ethnicity

Studies have shown that ethnicity plays a role in disease progression and severity [24–26]. A large cohort study investigating 53,065 heart failure patients showed that one-year mortality was lower in Black, Hispanic and Asian patients compared to White patients [27] and a meta-analysis has shown that Indian and Malay ethnic groups in South East Asian countries are at higher risk to develop metabolic diseases like diabetes [28–30]. A propensity-matched study done amongst coronary artery bypass graft patients showed that South Asian ethnicity had a survival benefit compared its predominantly White counterparts [31]. Our study has shown that even after removing the effect of ethnic variances in incidences of commonly studied comorbidities through multivariate logistic regression, ethnicity alone appears to be an independent predictor of one-year mortality. This could be due to some less studied genetic factors that result in Chinese ethnicity being a protective factor for peri-operative mortality but further studies would be needed to ascertain whether the differences in mortality rates are due to ethnicity affecting disease progression which in turn affect mortality, or if there are other direct genetic factors.

### Peripheral vascular disease

Our data shows that having chronic medical disease such as diabetes, hypertension and hyperlipidaemia have no significant effect on one-year mortality. However, our finding that peripheral vascular disease is significant for mortality suggests that poor control of these chronic medical conditions allowing progression to end organ dysfunction would then have an adverse impact on mortality. This reiterates the need for effective screening, treatment and follow up for chronic medical conditions.

## Conclusion

Even if patients do survive the immediate perioperative period, longer term outcomes are also relevant to their overall well-being and would influence their decisions regarding extent of care. This study should be a platform for larger scale studies to eventually develop a risk scoring system for risk stratification, counselling and surgical planning. This would help in patients’ care planning.

The study also reiterates the need for timely identification and treatment of pre-operative anaemia and malnutrition as well as smoking cessation. Although these factors have been previously shown to adversely affect 30-day mortality, we have shown that this extends beyond the immediate perioperative period into longer term outcomes.

We acknowledge the limitations of a retrospective study. However, as mortality is a rare and unpredictable outcome, it would be challenging to conduct such a study prospectively.

Furthermore, unlike studies done on large databases, the integrity of our data was ensured by having members of the research team manually verify the variables and outcomes against patients’ medical records to ensure accuracy.

## Figures and Tables

**Table 4:**
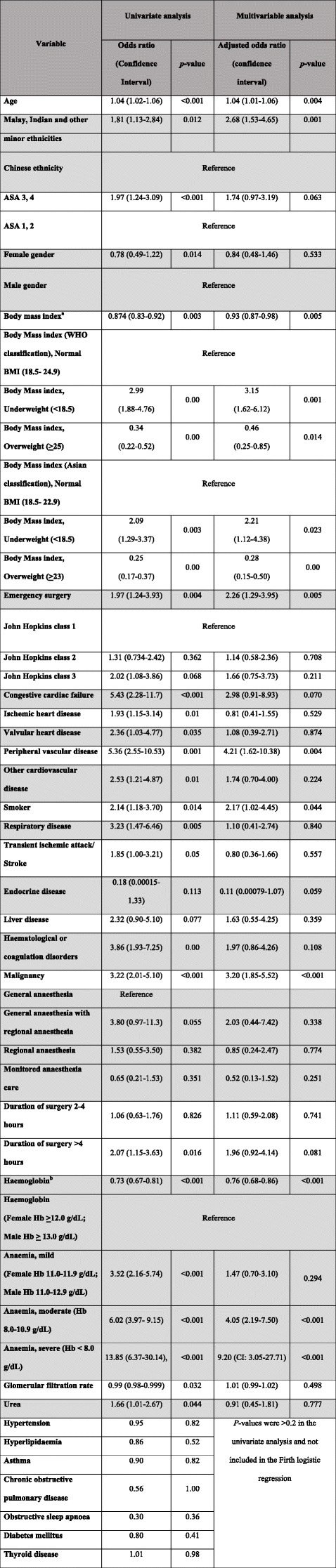
Table showing demographic, clinical and surgical data that were analysed

^a^For every 1 point increase in BMI

^b^For every 1 g/dL drop in haemoglobin

## Data Availability

All data generated or analysed during this study are included in this published article.

## Abbreviations

ASA: American Society of Anaesthesiologists physical status classification system
BMI: Body mass index
